# Elevated Concentrations of SERPINE2/Protease Nexin-1 and Secretory Leukocyte Protease Inhibitor in the Serum of Patients with Papillary Thyroid Cancer

**DOI:** 10.1155/2017/4962137

**Published:** 2017-01-31

**Authors:** Tomasz Stępień, Mateusz Brożyna, Krzysztof Kuzdak, Ewelina Motylewska, Jan Komorowski, Henryk Stępień, Hanna Ławnicka

**Affiliations:** ^1^Clinic of Endocrinological and General Surgery, Chair of Endocrinology, Medical University of Lodz, Pabianicka 62, 93-513 Lodz, Poland; ^2^Department of Immunoendocrinology, Chair of Endocrinology, Medical University of Lodz, Sterlinga 3, 91-425 Lodz, Poland; ^3^Clinic of Endocrinology, Chair of Endocrinology, Medical University of Lodz, Sterlinga 3, 91-425 Lodz, Poland

## Abstract

*Introduction*. SERPINE2 and secretory leukocyte protease inhibitor (SLPI) are proteins with anticoagulant properties which could promote solid tumor growth. However, their role in the pathogenesis of thyroid cancer has not been determined.* Materials and Methods*. The aim of this study was to assess serum SERPINE2 and SLPI concentrations in a group of 36 patients with papillary thyroid cancer (PTC) and a group of 19 subjects with multinodular nontoxic goiter (MNG). The control group (CG) consisted of 20 healthy volunteers. Blood samples were collected one day before surgery. Serum SERPINE2 and SLPI concentrations were measured using specific ELISA methods.* Results*. Significantly higher concentrations of SERPINE2 and SLPI were found in patients with PTC as compared with MNG and controls. Positive correlation was found between SERPINE2 and SLPI concentrations in PTC patients. The levels of SERPINE2 and SLPI did not differ significantly between MNG and healthy controls.* Conclusions*. Our results indicate that SERPINE2 and SLPI play a significant role in the development of papillary thyroid cancer and imply that the evaluation of serum concentrations of both anticoagulant molecules may be considered as additional marker for the differentiation of malignancies during the preoperative diagnosis of patients with thyroid gland tumors.

## 1. Introduction

Thyroid cancer (TC) is the most common endocrine malignancy, occurring in ~5%–10% of patients with a thyroid nodule [1]. Papillary thyroid cancer (PTC) is the most frequent TC subtype, accounting for more than 75% of all thyroid malignancies with 10-year survival of over 90% [2]. Several clinical, cytological, and molecular markers, including serum thyroglobulin, TSH-R mRNA, and thyroglobulin antibody levels, are well-known prognostic factors in patients diagnosed with PTC [3]. In addition, genetic alterations such as BRAF point mutations, RET/PTC rearrangements, or RAS point mutations also have prognostic value for papillary thyroid tumors [4]. Serpin peptidase inhibitor clade E member 2 (SERPINE2/protease nexin-1) and secretory leukocyte protease inhibitor (SLPI) are secreted serine protease inhibitors which are overexpressed in a number of cancers and involved in tumor formation [5–7]. Both inhibitors enable primary tumor cells to form vascular-like networks and facilitate perfusion and are often overexpressed in various cancer tissues including lung, brain, head/neck, and breast cancers [8–11]. However, the oncogenic potential of SLPI in thyroid cancer remains unknown. Therefore, the aim of the present study was to determine the potential role of SERPINE2 and SLPI in the pathogenesis of PTC and to identify whether SERPINE2 and SLPI serum concentrations might be considered additional prognostic factors of malignancy in patients diagnosed with PTC.

## 2. Materials and Methods

### 2.1. Study Design and Patient Characteristics

Fifty-five patients aged from 18 to 75 years (53.72 ± 12.62) (mean ± standard error of the mean) and treated by surgery in the Clinic of Endocrinological and General Surgery, Copernicus Memorial Hospital, Lodz, Poland, between 2012 and 2015 were enrolled into the study. The examined group was composed of 36 subjects diagnosed with PTC (PTC group) and 19 patients suffering from multinodular nontoxic goiter (MNG group).

Selected cases were diagnosed by fine-needle biopsy and confirmed by postoperative histopathologic examination. Other thyroid gland pathologies were excluded on the basis of familial and clinical history, clinical examination, ultrasonography, and thyroid function tests (aTPO, aTG TSH, fT3, fT4, and calcitonin serum concentrations). The control group (CG) included 20 healthy, age-matched volunteers with no history of any thyroid disease confirmed by clinical, hormonal, thyroid ultrasound scan and the presence of thyroid autoantibodies. Demographic and clinical characteristics of the examined groups and healthy controls are presented in Table 1. All patients diagnosed with PTC were treated with total thyroidectomy, and therapeutic neck dissection was performed with standard indications. The MNG patients group was treated by bilateral subtotal thyroidectomy. The final histopathological diagnosis in surgically treated patients was as follows: 36 cases of malignant primary papillary carcinomas of the thyroid, including six histological subtypes, such as conventional, follicular variant, tall cell, columnar cell, and diffuse sclerosing thyroid carcinoma. Histopathological diagnosis and clinical staging of patients included in the study are presented in Table 2. According to the 2010 TNM system edition by the Union for International Cancer Control, twelve PTC patients had stage I and another twelve patients had stage II, and ten patients presented with a PTC classified as stage III, whereas two subjects were classified as stage IVA [12]. The multinodular nontoxic goiter group was comprised of 19 cases with benign postoperative histopathological diagnoses, including 8 follicular adenomas and 11 adenomatous nodules.

The project was approved by the Bioethics Committee of the Medical University of Lodz.

### 2.2. Measurements of SERPINE2 and SLPI Serum Levels by ELISA

Blood samples were collected from the antecubital vein between 7:00 and 8:00 am after an overnight fast, one day before surgery. Blood samples were processed within one hour after collection and serum-aliquoted and stored at −80°C until analysis. Determinations of SERPINE2 and SLPI concentrations were evaluated using enzyme-linked immunosorbent assay (ELISA) kits (Shanghai Sunred Biological Technology Ltd.), following the manufacturer's instructions. All measurements were taken in duplicate and averaged.

### 2.3. Statistical Analysis

Results were presented as mean ± standard error of the mean (SEM). The Shapiro-Wilk test was applied to analyze the data distribution. ANOVA followed by Fisher's protected Least Significant Difference was used to calculate differences between investigated groups; *p* < 0.05 was considered significant. The independent relationship between serum SERPINE2 and SLPI concentration was examined using Pearson's linear correlation analysis. All statistical analyses were performed using the StatSoft statistical software v. 10.0. (Statistica PL).

## 3. Results

**Figure 1:**
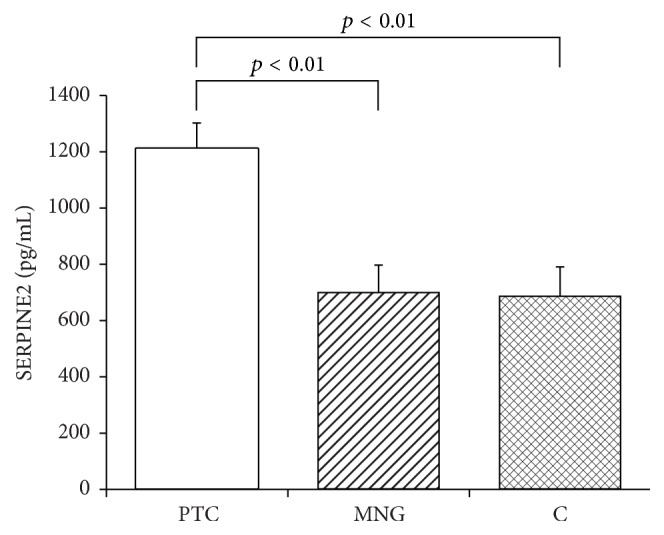
Mean serum concentration of SERPINE2 (pg/mL) in patients diagnosed with papillary thyroid cancer (PTC) or multinodular goiter (MNG) and in healthy volunteers (C). Bars represent mean ± SEM.

**Figure 2:**
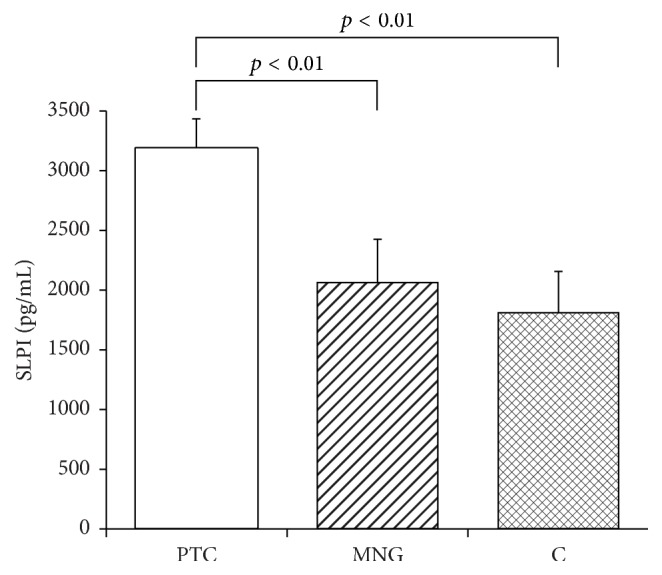
Mean serum concentration of SLPI (pg/mL) in patients diagnosed with papillary thyroid cancer (PTC) or multinodular goiter (MNG) and in healthy volunteers (C). Bars represent mean ± SEM.

The quantitative determination of the SERPINE2 and SLPI concentrations in the serum and the statistical evaluation of these results is presented in Figures 1 and 2. The mean serum level of SERPINE2 in PTC patients (1208.1 ± 93.9 pg/ml) was significantly higher than that obtained in the MNG group (700.1 ± 98.7 pg/ml: *p* < 0.01) or controls (686.8 ± 104.6 pg/ml; *p* < 0.01). SERPINE2 levels in patients diagnosed with MNG did not differ significantly from those of healthy controls (*p* > 0.05). Also the mean SLPI serum level was significantly higher in PTC patients (3181.6 ± 252.5 pg/ml) than the MNG group (2060.8 ± 363.3; *p* < 0.01) and controls (1806.7 ± 346.9 pg/ml; *p* < 0.01). The mean serum SLPI concentration in MNG diagnosed patients did not differ from that of the control group (*p* > 0.05).

A strong positive correlation was found between serum concentrations of SERPINE2 and SLPI (*r* = 0.906; *p* < 0.001) in the PTC group.

## 4. Discussion

The serpins (serine proteinase inhibitors) comprise a structurally related superfamily of proteins (350–500 amino acids in size) of the chymotrypsin family. Human serpins are involved in a diverse set of biological functions. While some serpins demonstrate biochemical functions which appear to be associated with proteinase inhibition, others do not, or their role in the biological process has not been defined.

One of the better known and more studied members of serpin superfamily is SERPINE2 (serpin peptidase inhibitor clade E member 2), also known as protease nexin-1 (PN-1). This 43 kDa glycoprotein is the closest relative of plasminogen activator inhibitor-1 (PAI-1 or SERPINE1) and can inhibit the activities of various proteases including thrombin, urokinase, trypsin, or plasmin [13]. SERPINE2 is overexpressed in a large number of invasive/metastatic tumors including breast, prostate, pancreatic, colorectal, oral-squamous, and testicular cancers and is required for tumor growth and malignant progression [6, 10]. In particular, SERPINE2 is upregulated by BRAF signal transduction [10], the more common genomic instability found in PTC. BRAF mutations are strongly associated with the tall-cell variant of PTC, although they have been also presented in conventional and follicular variant [14]. Other variants of PTC are rare and BRAF mutation has not been investigated in these uncommon subtypes of PTC. However, the precise function of SERPINE2 in the genesis and progression of human thyroid cancers through upregulating cell division and proliferation still remains elusive. Secretory leukocyte protease inhibitor (SLPI), a 11.7 kDa serine protease inhibitor, belongs to the whey acidic protein four-disulfide core family with antibacterial and antifungal activity [15, 16]. SLPI has also been reported to play a role in cell proliferation, cancer progression, metastasis, and invasion [17, 18]. A recent study showed that SERPINE2 and SLPI are produced in cancer tissues and are overexpressed under tumorigenic conditions. Abnormal higher expression of both serine protease inhibitors can be often detected in various cancer tissues including lung, head/neck, and pancreatic cancers [11, 19]. SLPI mRNA was overexpressed in serosa invading gastric cancer cells, and the cell migration and invasion rate was significantly increased in SLPI overexpressing gastric cancer cell line [20]. In addition, the estimation of serum SLPI levels in patients has been used to improve specificity of ovarian cancer diagnosis [21].

SERPINE2 and SLPI have also been postulated as new molecular targets in the therapy of certain cancers [22–25]. However, the role of SERPINE2 and SLPI in thyroid gland oncogenesis has yet to be studied. The present study is the first to identify elevated serum concentrations of SERPINE2 and SLPI in patients diagnosed with all histopathological variants of PTC. In the group of MNG patients, with benign tumors, peripheral blood serum levels of SERPINE2 and SLPI did not differ from those seen in healthy controls. These findings suggest that increased concentrations of both serine protease inhibitors are associated with an early stage of thyroid epithelial cells oncogenesis [26]. The transversion of early-stage thyroid tumors to more aggressive and invasive form is in agreement with clinical and experimental data of other authors, who showed that the cellular overexpression SERPINE2 and SLPI may augment solid tumors cell proliferation, remodeling of cytoskeleton, and the acquisition of a migratory phenotype [5, 27, 28]. In addition, the cellular overexpression of SERPINE2 and SLPI may suggest higher aggressiveness of solid tumors and poor outcome of the disease [29, 30]. The strong positive correlation between serum concentrations of SERPINE2 and SLPI identified in patients diagnosed with PTC in the present study may further confirm the hypothesis that both serine protease inhibitors may be involved in thyroid gland oncogenesis.

However, the mechanism responsible for the elevated serum concentration of SERPINE2 and SLPI in PTC patients remains unknown and further experiments are hence necessary to clearly identify the molecular mechanisms associated with SERPINE2 and SLPI during the morphological transformation between human normal thyroid epithelial cells and neoplasms.

## 5. Conclusion

Our findings indicate that the estimation of SERPINE2 and SLPI serum concentration may allow the preoperative differentiation of malignant and benign tumors of the thyroid gland. As the number of patients in our investigated groups was quite small, future studies with larger groups will be needed to verify these findings and identify the molecular mechanisms behind SERPINE2 and SLPI activity as a part of larger clinical trials.

## Figures and Tables

**Table 1:** Demographic and clinical characterization of the patients diagnosed with PTC or MNG and healthy controls (C).

Group	Number of patients	Gender F/M	Age (years) Mean ± SEM	Clinical status
Control	20	10/10	55.15 ± 9.80	Euthyreosis
PTC	36	20/16	52.73 ± 11.20	Euthyreosis
MNG	19	10/9	54.71 ± 10.23	Euthyreosis

**Table 2:** Histopathological diagnosis and clinical staging of 36 PTC patients included in the study, recommended by the 2010 TNM system edition by the Union for International Cancer Control [12].

PTC variants (*n* = number of patients)	Stage of PTC			
	I	II	III	IVA
Conventional *n* = 14	7	3	4	0
Follicular variant *n* = 12	5	4	3	0
Tall cell *n* = 3	0	1	1	1
Columnar cell *n* = 2	0	0	1	1
Diffuse sclerosing *n* = 2	0	1	1	0
Clear cell *n* = 3	0	3	0	0
